# Incidence and outcome of laryngeal edema and rhabdomyolysis after ingestion of black rock

**DOI:** 10.1186/s12245-023-00577-y

**Published:** 2024-01-02

**Authors:** Aml Ahmed Sayed, Abdelrahman Hamdy Abdelrahman, Zein Elabdeen Ahmed Sayed, Marwa Ahmed Abdelhameid

**Affiliations:** 1 Internal Medicine Department, Faculty of Medicine, Aswan University, Aswan, Egypt; 2 Internal Medicine Department, Faculty of Medicine, Assiut University, Assiut, Egypt

**Keywords:** Black rock, Laryngeal edema, Rhabdomyolysis, Acute liver injury, ECG changes

## Abstract

**Background:**

Black rock, Kala Pathar or ParaPhenyleneDiamine (PPD), is an aromatic amine widely used as a hair dye ingredient and is also used in textile industries. However, when ingested, PPD is highly toxic resulting in angioneurotic edema, rhabdomyolysis, acute kidney injury, toxic hepatitis, and myocarditis with a high mortality rate. This study aimed to evaluate the incidence and outcome of laryngeal edema and rhabdomyolysis after ingestion of PPD.

**Patients and methods:**

The current research was a cross-sectional study that was conducted at Aswan University Hospital, Aswan, Egypt, from December 2021 to December 2022. It consisted of 100 people who attempted suicide by ingesting black rock. All patients underwent general examinations and investigations, including complete blood count, urea, creatinine, creatine phospho kinase, alanine aminotransferase, aspartate aminotransferase, calcium, uric acid, phosphorus, urine analysis, and electrocardiography.

**Results:**

The current study consisted of 15 males and 85 females; the most common presentation was stridor (88%) followed by muscle weakness (50%). Twelve percent of patients with stridor required tracheostomy while 14% required tracheal intubation. Regarding the complications of PPD ingestion, the incidence of hepatic injury was (97%) and acute kidney injury (14%) five of them required hemodialysis, with a mortality rate of 13%. Cardiac arrhythmias were noticed in the form of sinus tachycardia (24%), sinus bradycardia (3%), atrial fibrillation (5%), ventricular fibrillation (6%), and ventricular tachycardia (7%). Our study found a significant positive correlation between creatine phosphokinase, muscle weakness, and acute kidney injury (*P* = 0.005). Whereas a significant positive correlation was noted between stridor, hospital stay, and mortality rate (*P* = 0.000), (*P* = 0.003), respectively. Moreover, a significant positive correlation was found between tracheotomy, mortality rate, and hospital stay (*P* = 0.000).

**Conclusion:**

PDD toxicity is more frequent in younger females. The intoxication from the black rock is increasingly used in suicide attempts and vital organs are usually affected especially the kidney, liver, and heart causing morbidity and mortality.

## Introduction

Suicide is a prevalent, avoidable healthcare issue in developed and developing nations; the suicide prevalence has increased by 60% over the past 50 years resulting in one million suicides annually. Most suicide cases seen in emergency departments are caused by self-poisoning [1], it is estimated that around 20% of global suicides are due to pesticide self-poisoning most of which occur in rural agricultural areas in low and middle-income countries, other common methods of suicide are hanging and firearms [2]. Because of its lower cost, high level of toxicity, and ease of availability, ParaPhenyleneDiamine (PPD) is now a common method of suicide particularly in the countries of East Africa, the Indian subcontinent, and the Middle East [3].

**Fig. 1:**
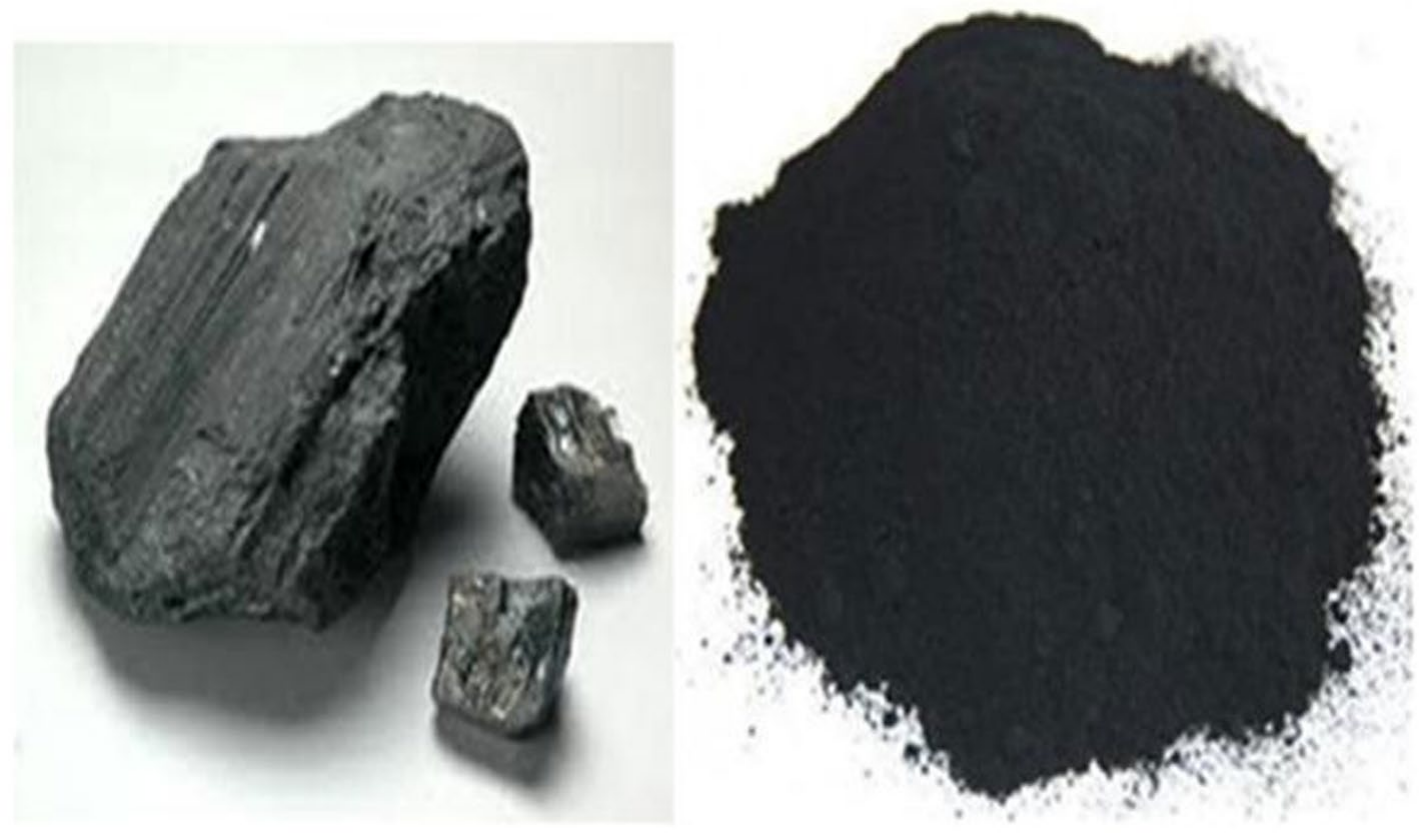
Black stone dye (before and after crushing)

PPD is widely present in Kala Pathar (black stone); once crushed it is typically mixed with henna (Lawsonia Alba) which is then used to color hair or stain soles and palms [4], it is marketed as a black stone dye in Egypt, as presented in Fig. 1. The use of hair dyes can be traced back to 4000 B.C. when the hair on Egyptian mummies was dyed with henna [5].

PPD when ingested is highly toxic to the muscular, respiratory, renal, hepatic, and cardiac systems; its toxicity is mainly due to the inhibition of cellular oxidation [6], and its effect largely relies on the dosage consumed; the lethal dosage is estimated to be between 7 and 10 g. PPD generates local and systemic toxicity when administered topically and/or ingested [7]. The initial report of systemic PPD poisoning was informed in 1924 and included a hairdresser who became toxic after receiving the dye [8].

Angioedema which can cause upper airway obstruction and difficulty breathing, rhabdomyolysis, acute kidney injury, acute hepatitis, myocarditis, convulsions, shock, and even death are all significant clinical symptoms of PPD ingestion [9]. Angioneurotic edema is the main presenting symptom of PPD toxicity to the Emergency department (ED) and it is the primary reason for death within the first 24 h, while acute kidney injury is the main complication occurring within the first week [10]. The mechanism involved in the development of rhabdomyolysis by PPD is by promoting calcium release and leakage of calcium ions from the smooth endoplasmic reticulum followed by continuous contraction and irreversible change in structure of the muscle. Rhabdomyolysis is the main cause of ARF and hypovolemia, and the direct toxic effects of PPD or its metabolites on the kidneys also contribute [11].

Due to the lack of a particular antidote early detection and supportive measures are the cornerstones of management. Immediate tracheostomy is the main line of treatment followed by forced alkaline diuresis, steroids, and antihistamines, in circumstances where renal failure develops dialysis is required [12].

Low mortality rate and incidence of complications among individuals who attempted suicide by ingesting black rock as a result of early interventions were the primary outcomes of the study.

## Patients and methods

### Study design, setting, and patients

The present research is a cross-sectional study involving 100 individuals from the emergency department, intensive care unit (ICU), and internal medicine department at Aswan University Hospital (a Middle Eastern community in Egypt), from December 2021 to December 2022. We used Steven K. Thompson’s equation to calculate the sample size.

#### Inclusion criteria

- Patients above 14 years ingested black rock
- Patients ingested black rock (suicidal-accidental)

#### Exclusion criteria

- Other causes of rhabdomyolysis(diabetes, drugs, thyroid disease, and trauma).
- Other causes of laryngeal edema (inhaled chemicals, fumes, drugs).
- Patients below 14 years old.
- Preexisting comorbidities (diabetes, hypertension, cardiovascular disease, chronic kidney disease, thyroid disease, and malignancy)

### Data collection

The following data were collected from each qualified patient: full medical history, including name, age, sex, accidental or suicidal, and the quantity of ingested dye and the duration of ingestion. A clinical examination was performed to examine temperature, blood pressure, heart rate, respiratory rate, urine output, urine color, stridor, and muscular weakness. Moreover, complete blood count (CBC), urea, creatinine, serum sodium and potassium, creatine phospho kinase (CPK), alkaline phosphatase, alanine aminotransferase (ALT), aspartate aminotransferase (AST), serum calcium and phosphorus, serum uric acid, arterial blood gases (ABG), urine analysis, and electrocardiography (ECG) were among the laboratory tests performed. The hospital stay duration, mortality rate (recovery-death), and monitoring of any complications or procedures such as tracheostomy and its types, duration and intubation, and hemodialysis were studied.

### Study outcomes

Low mortality rate and incidence of complications among individuals who attempted suicide by ingesting black rock as a result of early interventions were the primary outcomes of the study. The secondary outcomes were the correlation between complications of black rock ingestion and mortality in these individuals.

### Ethical statement

We certify that the study adheres to national and global ethical guidelines. Regarding any of the following: physical, psychological, social, legal, economic, or additional variables, there are no potential hazards for research participants. Those who participated were given a thorough explanation of the study goals, methodology, risks, and advantages. consent A written informed was taken from participants in the study. Our study was conducted in accordance with the Declaration of Helsinki for studies on human subjects. Moreover, the study has been reviewed and authorized by the ethics committee of the Faculty of Medicine, Aswan University, Egypt (Asw.uni./523/3/21).

### Statistical analysis

Data were collected, revised, coded, and analyzed using the Statistical Package for Social Science (IBM SPSS) version 20. The qualitative data were presented as numbers and percentages, while quantitative data were presented as mean, standard deviations, and ranges when their distribution was parametric. A comparison between the two groups with qualitative data was performed using the chi-square test, Fisher’s exact test was used instead of the chi-square test when the expected count in any cell was less than five. The comparison between two independent groups with quantitative data and parametric distribution was conducted using an independent *t*-test. The comparison between two independent groups with quantitative data and non-parametric distribution was performed using the Mann–Whitney Test. The confidence interval was set to 95%, and the margin of error accepted was set to 5%. Therefore, the *p* value was considered significant as the following: *P* > 0.05 = non-significant (NS), *P* < 0.05 = significant (S), and *P* < 0.001 = highly significant (HS). We used Steven K. Thompson’s equation to calculate the sample size, as shown in Table 1.

$$\textrm{n}=\frac{{\textrm{N}}^{\ast}\textrm{P}\left(1-\textrm{P}\right)}{\left(\left(\textrm{N}-{1}^{\ast}\left({\textrm{d}}^2/{\textrm{Z}}^2\right)\right)+\textrm{P}\left(1-\textrm{P}\right)\right.}$$

*n*: Sample size (100)

*N*: Population size (135)

*Z*: Confidence level at 95%(1.96)

d: Error proportion (0.05)

*P*: Probability (50%)

**Table 1:** Groups distribution according to sample size

	Female	Male	Total
Total	115	20	*N* = 135
%	85.0%	15.0%	100%
Sample size calculated	85	15	*n* = 100

## Results

The current study included 100 participants from Aswan University Hospital. Most patients were females (85%) with a mean age of 24.07 ± 6.51 years. The number of ingested packets ranged from one to six with a mean of 3.2 ± 1.5 packets (each packet contains 1 g of PPD) with an intake duration of one to four hours with a mean of 2.5 ± 1.01 h. All cases revealed dark urine (100%) with a mean urine output of 903 ± 343.3 mL.

**Fig. 2:**
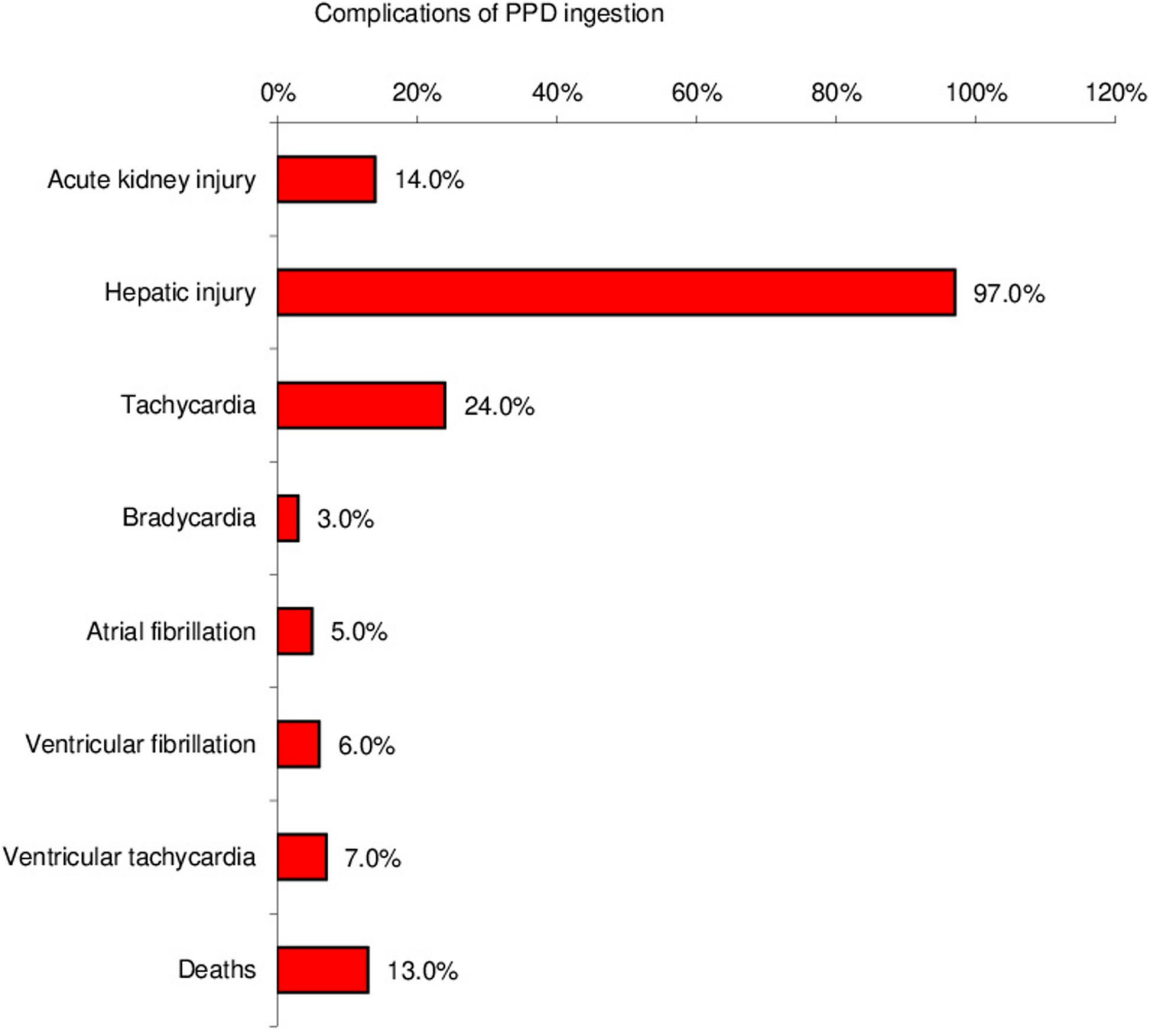
Complications of PPD ingestion

The most prevalent symptom was stridor (88%) followed by muscle weakness (50%) as a result of rhabdomyolysis. As shown in Fig. 2, the incidence of hepatic injury was (97%) and acute kidney injury (AKI) was (14%); five of them required hemodialysis with a mortality rate of 13%. Moreover, cardiac arrhythmias were noticed in the form of sinus tachycardia (24%), sinus bradycardia (3%), atrial fibrillation (5%), ventricular fibrillation (6%), and ventricular tachycardia (7%).

**Fig. 3:**
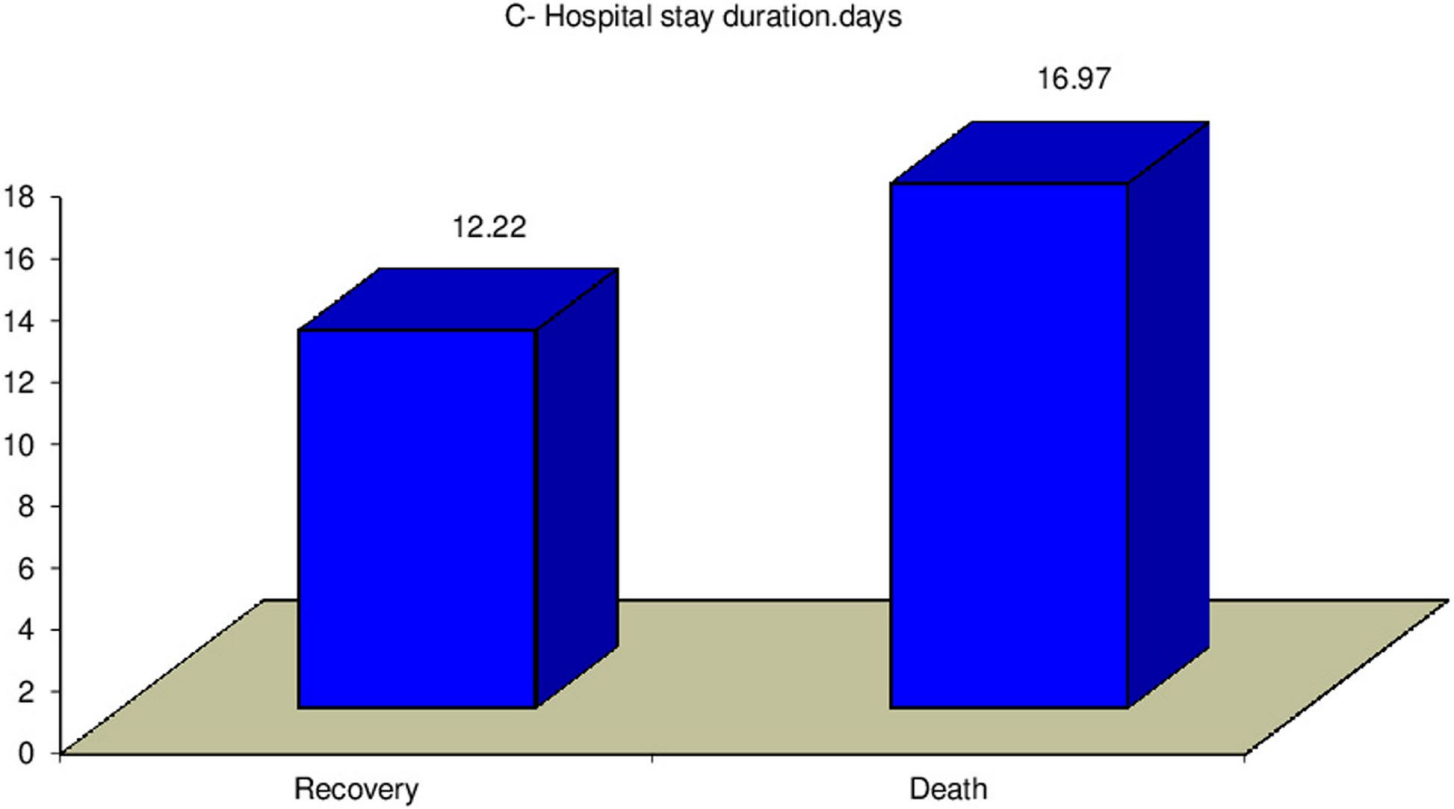
Relation between hospital stay duration and mortality rate

**Fig. 4:**
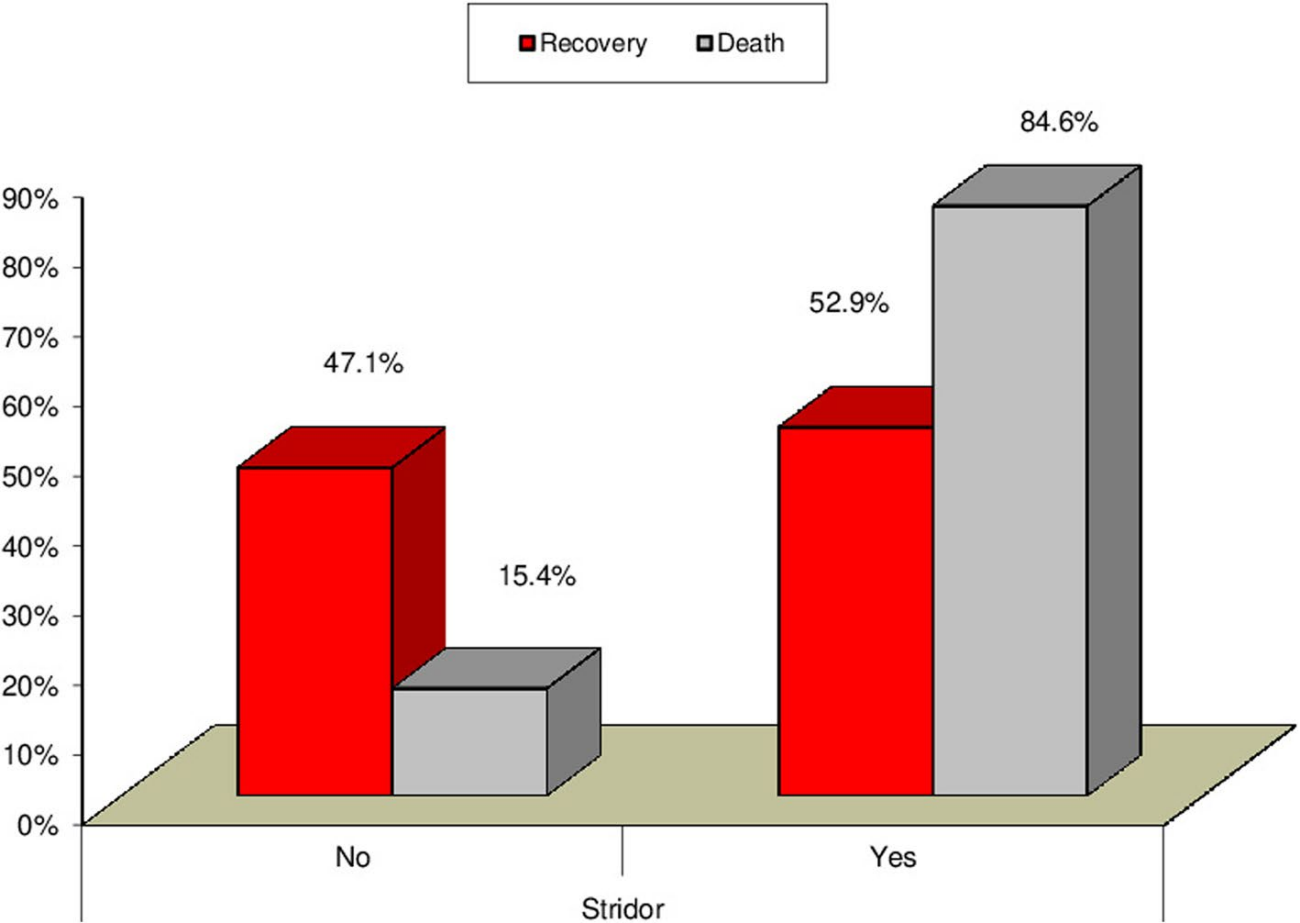
Relation between stridor and mortality rate

**Fig. 5:**
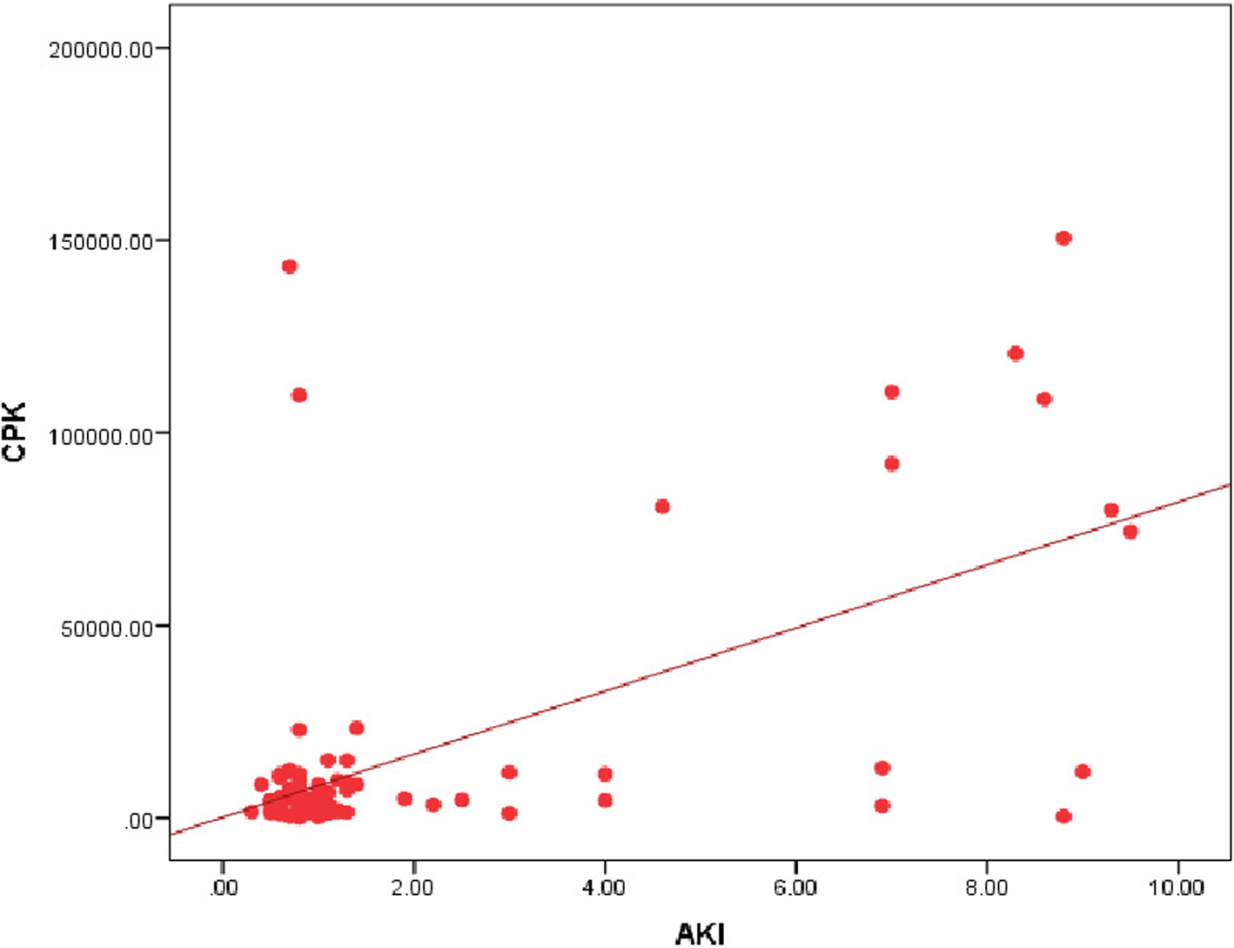
Correlation between CPK and AKI

**Fig. 6:**
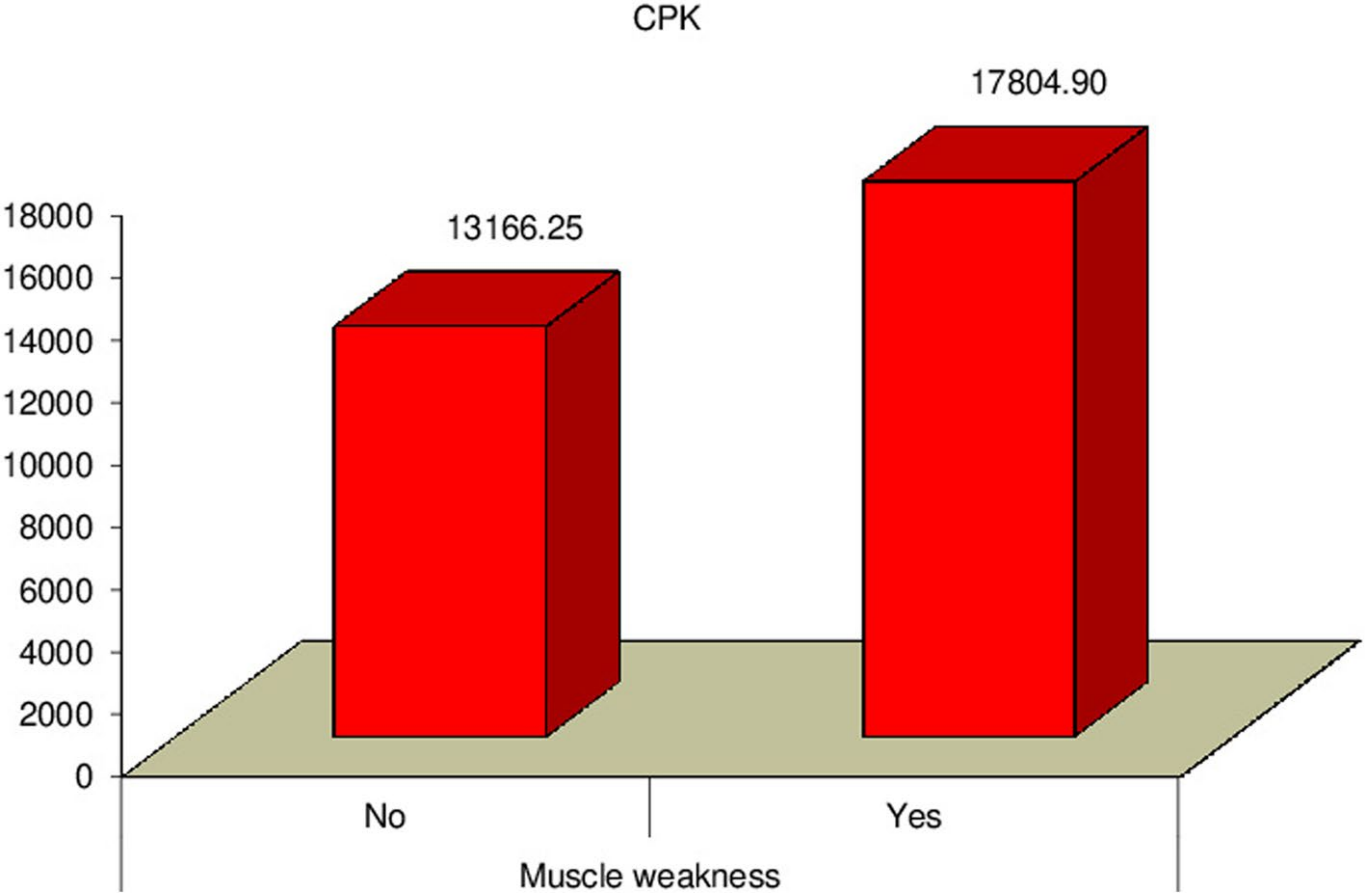
Relation between CPK and muscle weakness

**Fig. 7:**
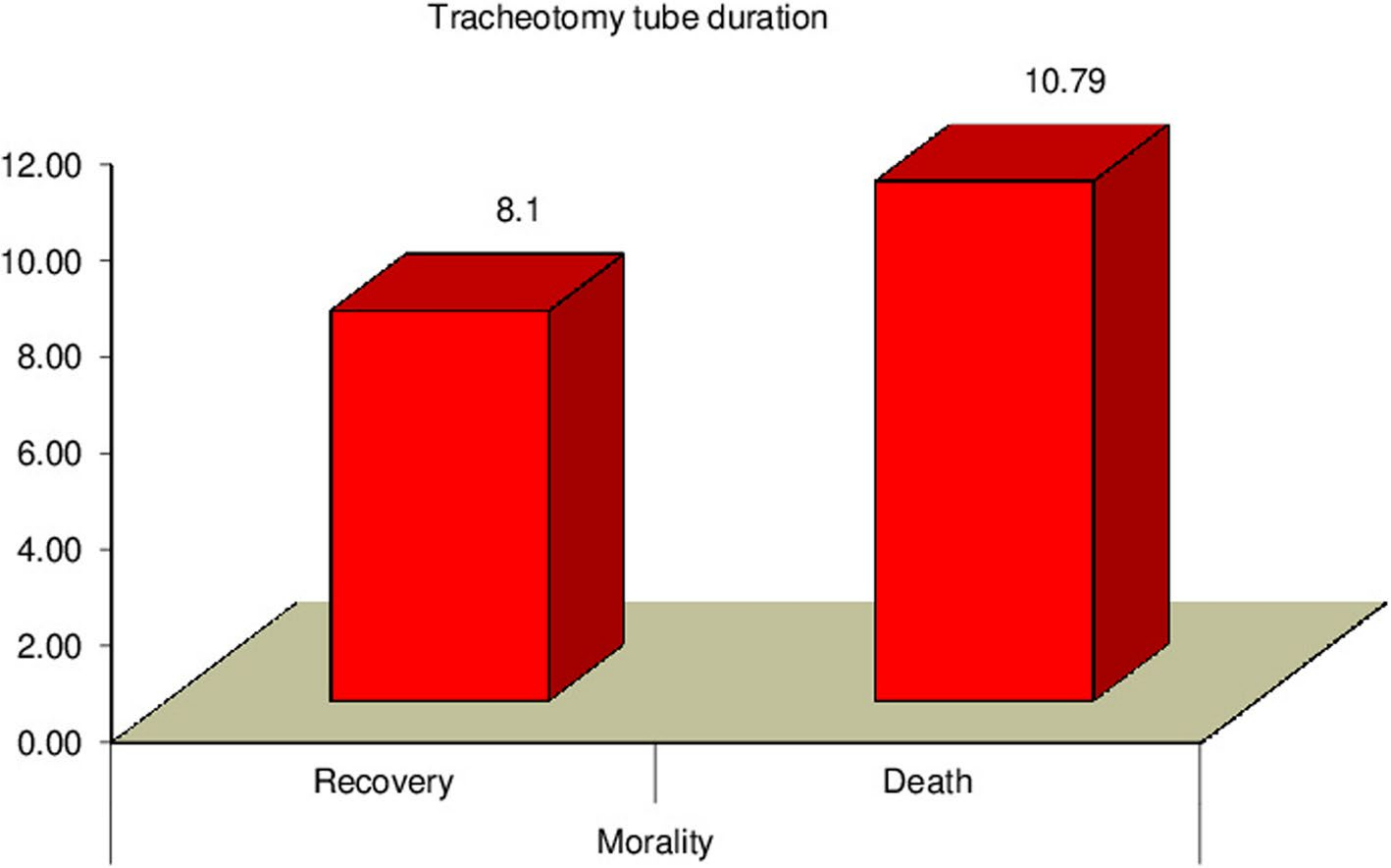
Relation between tracheostomy tube duration and mortality rate

**Fig. 8:**
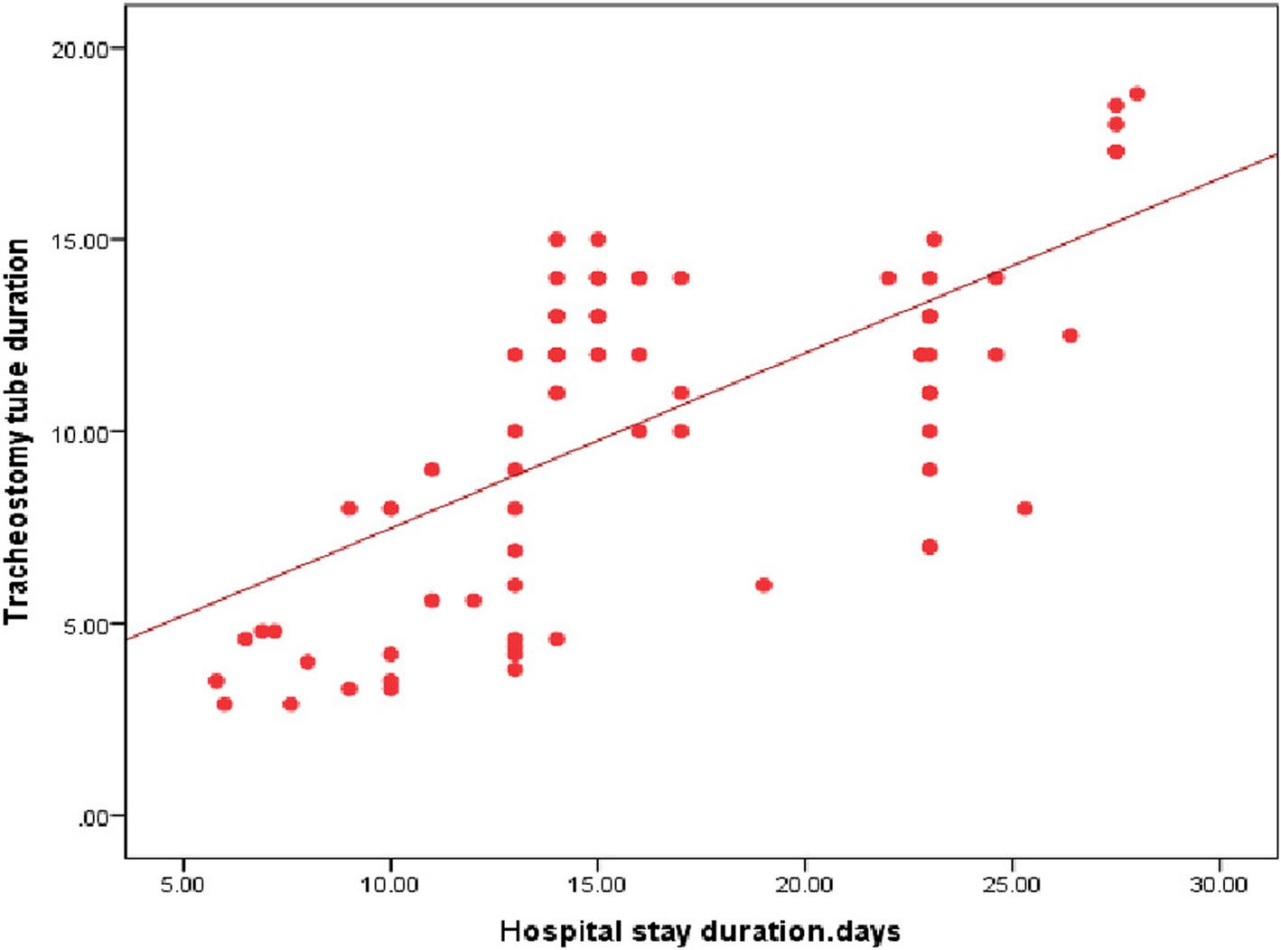
Correlation between tracheostomy tube duration and hospital stay

Our study revealed a significant relation between mortality rate, stridor, and hospital stay duration (*P* = 0.0007) (*P* = 0.0031) respectively, as presented in Figs. 3 and 4. Additionally, there was a significant relation between CPK, muscle weakness, and AKI (*P* < 0.01), also a significant relation was found between Tracheostomy tube duration, mortality rate, and hospital stay duration (*P* < 0.01), as presented in Figs. 5, 6, 7, and 8.

**Fig. 9:**
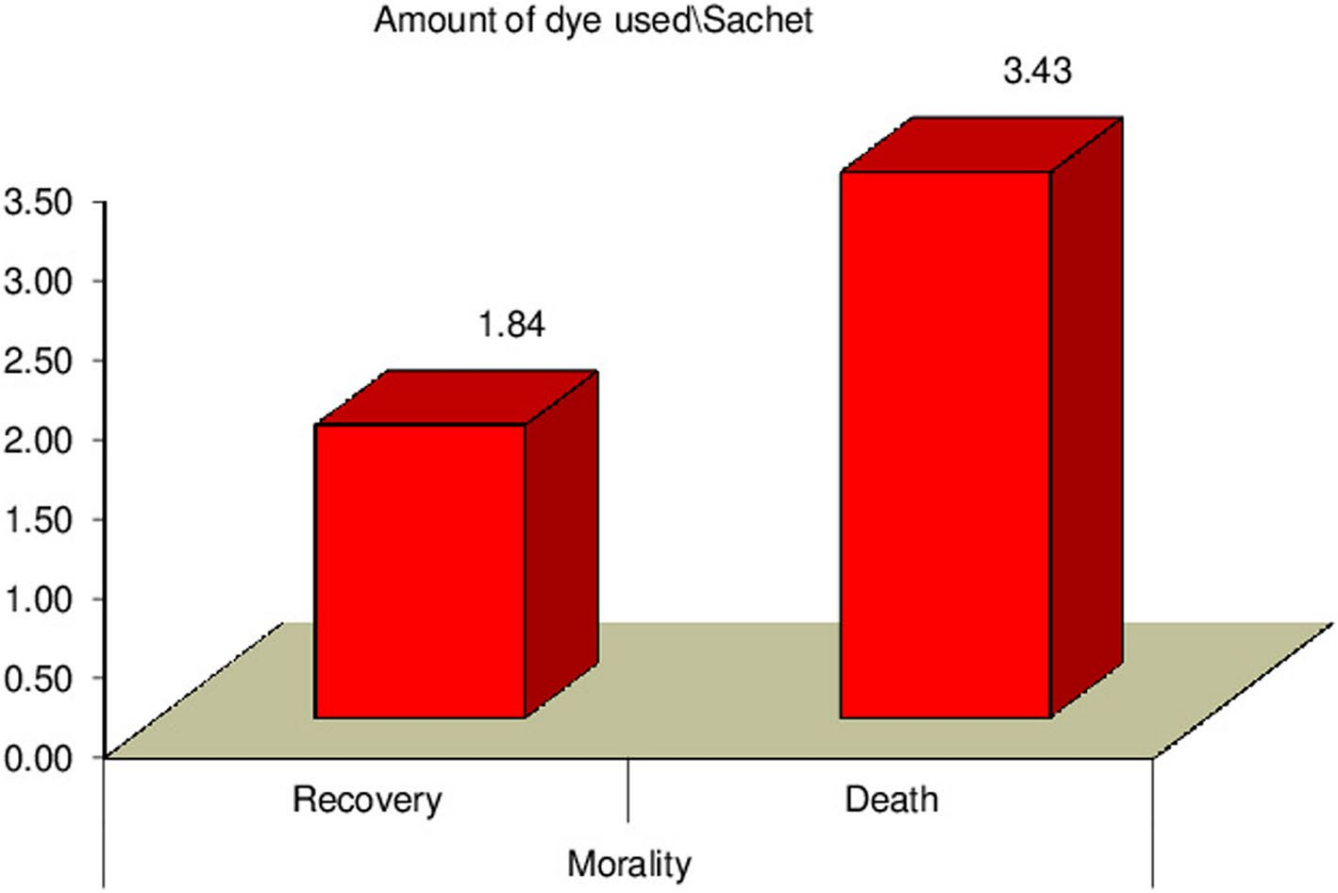
The relation between the amount of dye used\sachet and mortality rate

Finally, our study revealed a significant relation between the amount of ingested PPD, stridor, tracheal intubation, tracheostomy, hospital stay duration, and mortality rate (*P* < 0.01); additionally, we found a significant correlation between the amount of ingested PPD and lab investigations on admission as urea, creatinine, CPK, ALT and AST (*P* < 0.05), as shown in Tables 2 and 3 and Fig. 9.

**Table 2:** The relation between the amount of dye used\sachet and complications of PPD ingestion

		Amount of dye used\Sachet		Test value^a^	*P* value
		Mean ± SD	Range		
Stridor	No	1.91 ± 0.83	1–3	3.444	0.008^**^
	Yes	2.98 ± 1.03	1–5		
Intubation	No	2.08 ± 1.12	1–4	-3.807	0.003^**^
	Yes	3.24 ± 1.00	1–5		
Morality	Recovery	1.84 ± 0.99	1–4	15.729	0.000^**^
	Death	3.43 ± 1.58	1–6		
Tracheostomy	Yes	3.35 ± 1.39	1–5	9.179	0.003^**^
	No	1.89 ± 1.27	1–3		
ECG	NSR	2.25 ± 1.24	1–3	125.011	0.000^**^
	Tachycardia	1.61 ± 0.72	1–3	27.423	0.000^**^
	Bradycardia	1.75 ± 0.50	1–2	2.443	0.121
	AF	2.00 ± 0.25	2–4	5.151	0.025^*^
	VF	1.58 ± 0.36	1–4	5.263	0.019^*^
	VT	1.63 ± 0.16	1–5	6.488	0.012^*^

*NSR* normal sinus rhythm, *AF* atrial fibrillation, *VF* ventricular fibrillation, *VT* ventricular tachycardia

*P*-value > 0.05: Non significant (NS); *P*-value < 0.05: Significant (S)*; *P*-value < 0.01: highly significant (HS) **

^a^Independent *t*-test

**Table 3:** Correlation between amounts of dye used \Sachet with lab investigations on admission and hospital stay duration

	Amount of dye used\sachet	
	*r*	*P* value
Urea	0.463**	0.000^**^
Creatinine	0.333**	0.001^**^
CPK	0.207*	0.042^*^
ALT	0.296**	0.003^**^
AST	0.396**	0.000^**^
Hospital stay duration\ days	0.542**	0.000^**^

*CPK* creatine phosphokinase, *ALT* alanine aminotransferase, *AST* aspartate aminotransferase

*P*-value > 0.05: Non significant (NS); *P*-value < 0.05: Significant (S)*; *P*-value < 0.01: highly significant (HS) **

## Discussion

PPD is an organic compound; it is a white solid that changes color into black when exposed to oxygen. The formation of PPD oxide derivatives such as benzoquinone diimide which is responsible for the destructive necrosis of muscle cells, rhabdomyolysis, myocarditis, and angioneurotic edema, also has fatal effects on different organs [13]. After the ingestion of PPD complex reactions occur resulting in oxidation of the substance with the production of several intermediate compounds nevertheless, the primary product is Bondrowski’s base which is allergenic, poisonous and mutagenic [14].

The current study included 100 participants; most of them were females (85%) with a mean age of 24.07 ± 6.51 years. The most prevalent symptom was stridor (88%) followed by muscle weakness (50%). All patients with stridor received an intravenous (IV) steroid at the time of presentation and Ear, Nose, and Throat (ENT) consultation was done urgently for the possibility of early intervention to decrease the incidence of complications (upper airway obstruction and death). Therefore only 12 of them required tracheotomy and 14 required tracheal intubation. As the excretion of Black rock occurs through the kidney, all patients had dark urine and received fluid resuscitation to decrease the incidence of AKI as a result of rhabdomyolysis or through the direct toxic effect of PPD on the kidney.

As mentioned above, the majority of the participants in the current research are females (85%); as the PPD is typically mixed with henna which is then used to color hair or stain soles and palms which is very common in a Middle Eastern community in Egypt and it is widely used among younger aged female, so propensity and gender ingestion is more in this age group of. The average age of the participants is 24.07 ± 6.51 years; all of the individuals ingested the dye in an attempt to commit suicide, as no cases presented with accidental ingestion over the period of the study. These findings were identical to numerous researches, including Arif et al. [15], who reported that (78%) were females in comparison (22%) were males in their study. However, the average age of the cases was 23.98 ± 7.68 years, suicide was the most frequent cause of PPD toxicity occurring in 92 (92%) followed by homicidal and accidental causes 4 (4%) for each. Additionally, Hanif et al. [16] stated that a male-to-female ratio of 1:18 indicated that females were disproportionately affected by this toxin. The findings of their study demonstrate that young people (22.08 ± 6.42 years) are predominantly affected by Kala-Pathar poisoning.

Our study revealed that stridor(88%) and muscle weakness(50%) were the two most prevalent symptoms. Kallel et al. [17] stated that cervicofacial edema (79%), chocolates-brown urine (74%), upper respiratory tract edema (68.4%), oliguria (36.8%), muscular edema (26.3%), and shock (26.3%) were the most prevalent clinical signs in 19 persons with PPD intoxication in Tunisia throughout 6 years. Furthermore, Kallel et al. [17] reported that cervical orofacial angioedema and dysphagia caused by localized mucosal irritation and a systemic allergic response typically require an emergency tracheostomy that begins within 6 to 8 h in 80% of cases. Bhagavathulaet al [18]. found that adults and children exhibited flaccid paraplegia, palatopharyngeal paralysis, and laryngeal paralysis. According to Jain et al. [19], PPD toxicity is frequently accompanied by rhabdomyolysis and acute kidney failure which significantly increase morbidity and mortality rates which have been documented to be as elevated as 23.92% in India. According to the findings of Sultan et al. [20], rhabdomyolysis was observed in 80% of patients, with 40.5% experiencing acute renal failure.

Regarding the complications of PPD ingestion, our study revealed a 97% incidence of hepatic injury and a 14% incidence of AKI; five of these patients required hemodialysis. This is consistent with the findings of Khaskheli et al. [21], who discovered that all patients had elevated liver enzymes with significantly higher AST levels, and Sultan et al. [20], who reported an elevation of ALT/AST levels, with 38% of their patients had abnormal kidney function.

Furthermore, we observed a significant relationship between CPK, muscle weakness, and AKI. Accordingly, Khaskheli et al. [21] found that CK levels are significantly increased. In the study by Asghar et al. [22], the toxic effects of PPD on muscle result in rhabdomyolysis, which increases the risk of AKI and sudden cardiac death**.** Contrary to our result, Arif et al. [15] showed that 80% of the patients experienced acute kidney failure and 95% of them improved after receiving therapy, while 5% still had residual renal damage; This depends on the time of presentation and early intervention, at Aswan governorate suicidal attempt by PPD ingestion is very common and most cases presented early to ED and early interventions were done initially at ED in the form of fluid resuscitation, tracheostomy or tracheal intubation which results in a low incidence of AKI and mortality rate.

Moreover, cardiac arrhythmias were noticed in the form of tachycardia (24%), bradycardia(3%), atrial fibrillation (5%), ventricular fibrillation (6%), and ventricular tachycardia (7%). Our findings are supported by a study by Baril [23], who discovered that ECG shows a variety of changes including sinus tachycardia, bundles branch blocks, intraventricular conduction defects, atrial and ventricular premature complexes, atrial fibrillations, ventricular tachyarrhythmia, elevated or depressed ST segment and T-waves inversion. According to Sultan et al. [20], 90% of patients showed one or more ECG changes varying from ST-T changes to ventricular tachycardia indicating that the ECG in myocarditis can reflect transient abnormalities which are typically non-specific and happen in many other cardiac diseases.

Our study also revealed a significant relationship between stridor and mortality rate. Similarly, Namiranian et al. [24] demonstrated that upper respiratory tract obstruction (angioedema) which manifests with a hard swollen protruding tongue and edematous bull neck is one of the most serious symptoms and the leading cause of death in PPD intoxication.

The current study showed that the mortality rate of PPD toxicity was 13%, Hanif et al. [16], reported a mortality rate of 47.4%; this rate is very high but comparable to other studies carried out byKhuhro et al. [25], Asgharet al [22]., Haider et al. [10], and Tanweer et al. [26] reported that the mortality rate was 37.5%, 31.6%, 20%, and 12%, respectively. The high mortality rate may be due to the lack of antidote and timely interventions especially tracheotomy.

## Conclusion

ParaPhenyleneDiamine (Kala-Pathar) toxicity was higher in younger females; the hair dye poisoning is becoming a common suicidal poisoning. The most common presentation of PPD toxicity were cervicofacial edema, laryngeal edema, and rhabdomyolysis (the hallmarks of PPD poisoning). Hepatic injury, AKI, and cardiac arrhythmias were common complications of PPD toxicity, so its effect on vital organs was responsible for morbidity and mortality. There is no specific antidote for PPD toxicity; therefore, the mainstay of management is early recognition, interventions (tracheostomy, tracheal intubation, and hemodialysis), and supportive measures (fluid resuscitation and IV steroid) to decrease the incidence of complications and mortality rate.

We acknowledge that the present study has some limitations; therefore, further multi-center studies with large sample sizes are still needed to assess the complications of PPD toxicity, advanced line of management, and detection of specific antidote.

## Data Availability

The datasets used during the current study may be made available from the corresponding author upon reasonable request.

## Abbreviations

ABG: Arterial blood gases
AKI: Acute kidney injury
ALT: Alanine aminotransferase
AST: Aspartate aminotransferase
CBC: Complete blood count
CPK: Creatine phospho kinase
ECG: Electrocardiography
ED: Emergency department
ENT: Ear, nose, and throat
HS: Highly significant
ICU: Intensive care unit
IV: Intravenous
NS: Non-significant
PPD: Paraphenylenediamine
S: Significant
